# AI in SERS sensing moving from discriminative to generative

**DOI:** 10.1038/s44328-025-00033-2

**Published:** 2025-02-21

**Authors:** Steven M. Quarin, Der Vang, Ruxandra I. Dima, George Stan, Pietro Strobbia

**Affiliations:** Department of Chemistry, University of Cincinnati, Cincinnati, OH USA

**Keywords:** Biotechnology, Nanobiotechnology, Nanoscience and technology, Techniques and instrumentation, Imaging and sensing, Optical spectroscopy

## Abstract

This perspective discusses the present and future role of artificial intelligence (AI) and machine learning (ML) in surface-enhanced Raman scattering (SERS) sensing. Our goal is to guide the reader through current applications, mainly focused on discriminative approaches aimed at developing new and improved SERS diagnostic capabilities, towards the future role of AI in SERS sensing, with the use of generative approaches to design new materials and biomaterials.

## Introduction

The advancement of artificial intelligence (AI) over the last few decades has had an impact in multiple fields (e.g., security, industrial automation and healthcare) by increasing accuracy and efficiency^1–3^. AI systems consist of two major models: discriminative and generative. Discriminative models aim to identify patterns and classify data based on input information, whereas generative models aim to create new data, generating previously non-existent content. The remarkable speed of AI’s development and improvement are due to the rapid growth and understanding of machine learning (ML) approaches. ML is the portion of AI that enables computers to perform data-driven tasks. Furthermore, ML models can extract high-level knowledge from data via backpropagation and can autonomously improve when trained with more data. ML can extract information, data features, changes, and recognize patterns from large or complex data sets, and use them to build predictive performance models to make decisions or solve specific problems and tasks. This perspective aims to discuss how these AI/ML models are currently being used, and can be used in the future, to advance sensing based on surface-enhanced Raman scattering (SERS)^4–7^.

Raman spectroscopy is a powerful tool for the analysis of materials and biological samples, due to its spectra with high molecular information content. The inherently low signal of Raman spectroscopy makes it challenging to observe the signal of an analyte only present in trace amounts, including environmental contaminants or disease biomarkers. To this end, SERS can be used to amplify the Raman signal numerous orders of magnitude enabling such sensing applications. Specifically, SERS can be used for sensing based on two main principles: intrinsic and extrinsic sensing. Intrinsic SERS sensing (also referred to as label-free) consists in the direct observation of the signal of analytes on the surface of the SERS substrate, where the Raman signal of these species is amplified/enhanced. In extrinsic sensing, the SERS signal is used in a sensing mechanism that will transduce a receptor binding into a change in the specific signal. An example of this sensing principle is a SERS-based sandwich assay, where a SERS tag is immobilized on a surface due to the presence of a biomarker to indicate its presence. These SERS mechanisms are demonstrably a powerful tool for sensing, as shown by the many SERS sensing applications discussed elsewhere^8–10^. However, SERS analyses continue to suffer from several issues, specifically with interpretability of signal in complex samples, limitation in multiplexing capabilities due to peak overlap and variation in substrate fabrication and/or sample preparation. The combination of SERS with ML is emerging as a strategy to enhance SERS analysis overcoming these issues.

**Fig. 1:**
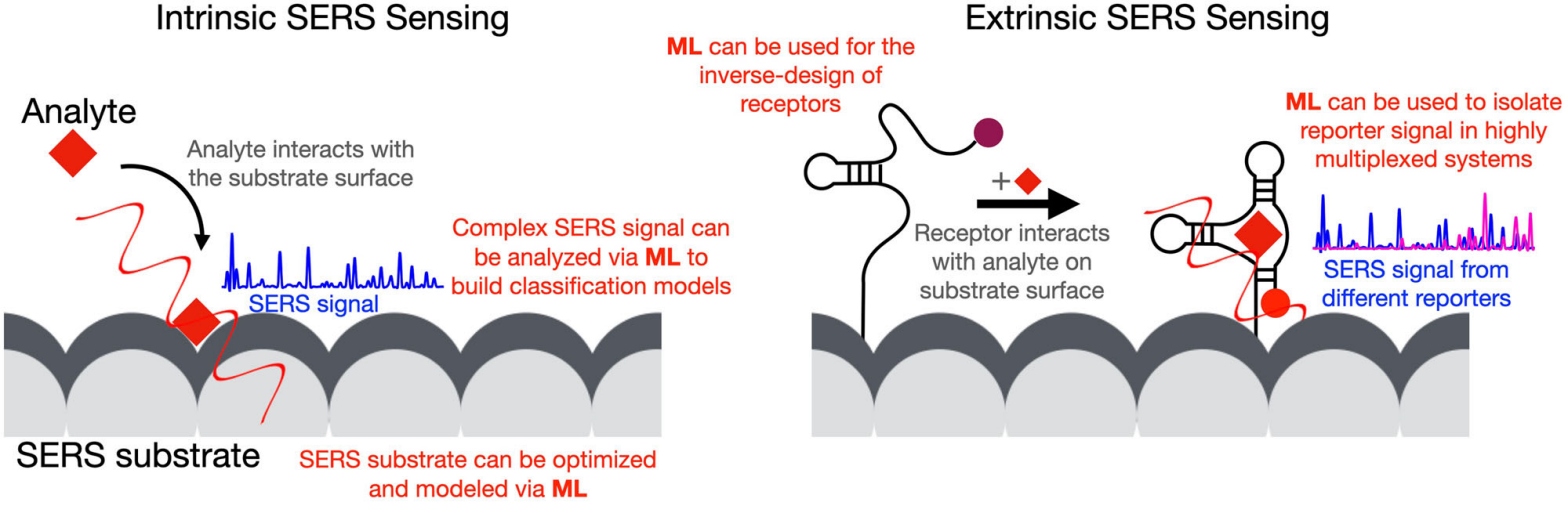
Schematic representation of the two mechanisms/principles of SERS sensing: intrinsic (left) and extrinsic (right). Intrinsic: analyte interacts with the SERS substrate surface and the SERS signal reflects the vibrational fingerprint of the analyte. Extrinsic: a SERS sensing mechanism involves a labeled receptor bound to the substrate surface -displayed here is the mechanism of a reagentless sensor-. When the analyte binds the receptor, a change in the position of the reporter/label translates in changes in the reporter SERS signal. In red, we highlight where ML can have an impact across SERS sensing.

This combined approach does not prescribe a unique, standard, methodology, but it has to be tailored to the specific SERS approach. The two SERS sensing principles (intrinsic and extrinsic) have distinct advantages and applications. Figure 1 shows the basic mechanism of each of the two principles. Intrinsic SERS sensing does not require specific knowledge or bioengineering to identify a target and obtain chemical information about the analyte. Chemical information can be correlated to a specific state of the sample (e.g., disease diagnosis in bioanalysis) and can also be used to gain additional knowledge about the system under analysis (e.g., molecular profiling). In extrinsic SERS sensing, the use of receptors offers improved sensitivity and specificity. This methodology is required to identify specific low-concentration targets in complex samples. Both intrinsic and extrinsic principles can harness the power of ML, each benefiting in a unique way. In intrinsic SERS sensing, the information-rich and complex spectra from biological samples can be analyzed using ML to identify specific indicators in the spectra. In extrinsic SERS sensing, the multiplexing power of SERS can be augmented by using ML to discriminate specific contributions in a complex mixture of reporters. Additionally, in both cases inverse design principle can be used to improve sensing capabilities based on material or receptor optimization. In this work, we discuss examples and opportunities for this combination (ML + SERS).

ML + SERS is a broad and complex topic treated in multiple reviews addressing different angles of this approach^11–13^. While our goal in this perspective is not to give a comprehensive review of ML techniques but rather to highlight the emergence of ML approaches in our field, as well as their versatile application to diverse aspects of SERS sensing. To aid the reader in following the perspective flow, this paragraph summarizes the key concepts of SERS + ML. Extensive discussion of these topics can be found elsewhere. In brief, Raman data are composed of series of sharp features that can be analyzed with multiple algorithms identifying variance or co-variance in the data. Widely used algorithms are principal component analysis (PCA) and partial least square (PLS), which can be used to reduce data dimensionality and help classification. PCA and PLS can be combined with other algorithms to perform classification in these lower-dimension spaces, such as linear discriminant analysis (LDA) and support vector machine (SVM). PCA is an unsupervised algorithm and can also be used to identify trends in the data to explore datasets and remove noise from them. Recently, the explosion of more complex ML algorithms has added many different algorithms to the toolbox. Examples are random forest (RF) and artificial neural networks (ANN) that can be used for the classification task of Raman data. Importantly, ML algorithms can also perform generative tasks, in addition to classification/discrimination. Generative adversarial networks (GAN) and variational autoencoders (VAE) are popular algorithms for generative AI. In the context of SERS sensing, these strategies could be used for tasks such as inverse design of materials and bio-receptors, at the forefront of our field. Herein, we discuss how some of these concepts/algorithms fit within the SERS biosensing field and where we think they could be used next.

### Current uses of machine learning in **intrinsic** SERS sensing

**Fig. 2:**
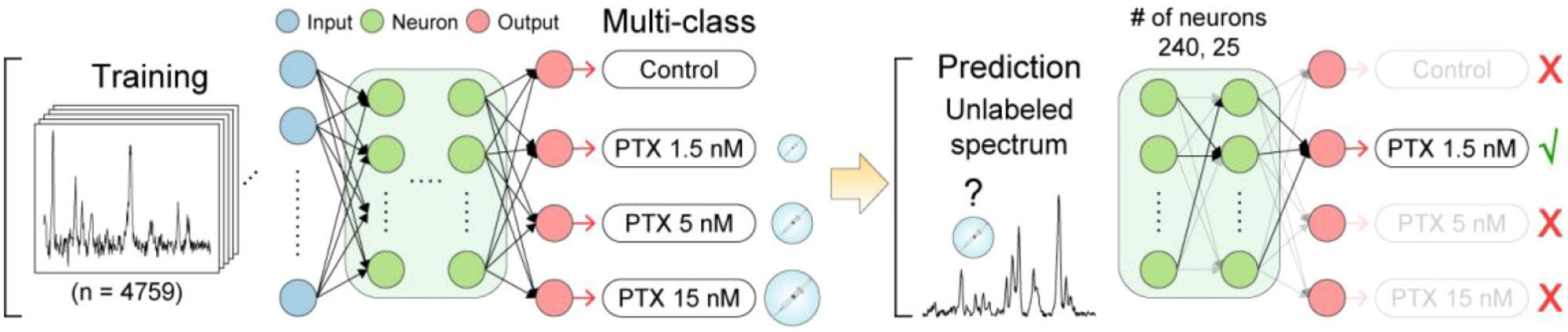
Schematic representation of artificial neural networks (ANN) applied for SERS classification cancer-cell treatments. A dataset of labeled spectra is used to train a classification model by selecting the important features and their combination to identify each class. Then, the model can be used by inputting an unlabeled spectrum into the model, which gives the correct classification as output. Adapted with permission from ref. ^15^ copyright 2022 American Chemical Society.

The most common use of ML in SERS sensing is to perform multivariate analysis of intrinsic SERS spectra. These analyses are a type of discriminative AI, where ML is used to find relations between a dataset and its known labels to create classification or regression models. Intrinsic SERS is ideally suited to be used in these models because of the rich information content of their spectra, which are often too complex to be manually analyzed but can contain multiple molecular signatures useful for classification. ML in intrinsic SERS has been the topic of multiple reviews, discussing how different ML models can be used to this end^7,11,13,14^. Fig. 2 shows the basic principle for this type of ML analysis^15^. As discussed in these reviews, most SERS models use principal component analysis (PCA) or partial least square (PLS)^13,14^. Interesting -but not comprehensive- applications of these processes are the discrimination of illicit drugs, the quantification of cortisol (an important stress biomarker), and the classification of mycobacteria using a lab-on-a-chip SERS device^16–18^. The first example is a microfluidic system integrated with an in-line SERS analysis of the sample. The authors use PCA to cluster/classify spectra of saliva samples containing either methamphetamine or heroin showing complete discrimination in the space based on the first two principal components^16^. The second example looks at cortisol concentrations and uses PCA to separate the spectral contribution due to the target analyte, which permits to quantify the latter^17^. The last example uses PCA and a linear discriminant analysis (LDA) method to create a hierarchical model that can distinguish between six different mycobacteria species^18^. These examples show that a straightforward dimensionality reduction approach (e.g., PCA) allows the extraction of important information from complex intrinsic SERS sensing spectra. Validation is a key component of any classification method and is often a challenge with these SERS-based dimensionality reduction methods, due to the absence of a clear relation between physical data and the model, especially for complex discriminant analysis. This issue can be even more of a challenge in more complex ML classification commonly labeled as “black box”, where the classification space (e.g., loadings) cannot be visualized.

While PCA and PLS are powerful ways to analyze high-dimensional SERS data and to map the dataset onto a simpler space (dimensionality reduction), these models are commonly considered challenging to interpret. Although the loadings obtained from PCA can be used to identify the specific SERS bands responsible for the classification, challenges in interpretation arise with the increased complexity of the discriminant analysis. To bypass this issue, our group has recently employed explainable AI models (XAI) for the classification of intrinsic SERS data^15^. Specifically, we measured spectra from commercial and clinical exosomes dried on SERS substrates. Next, we trained and validated a series of models to identify the most accurate model. Our results showed that bagging algorithm models (e.g., extra trees) gave the highest classification accuracy. While not outperforming PCA/PLS methods, the notable innovation in this work stems from the use of Shapley additive explanations (SHAP)^19^, an approach derived from Game Theory that reports the contribution of individual features to the predictions of the model, to bridge the gap between ML models and human comprehension. Through this process, we were able to report the specific Raman bands responsible for the classification, which provides several benefits: the ability to gain chemical insight, the increased confidence in using these models in the translation to real-world applications and the reduction of overfitting or model bias risk. These results agree with other reports where exosomes have been analyzed via SERS and classified via ML with accurate results^20–26^. An intriguing use of ML that avoids the limitations due to increased complexity is ML-based hypothesis testing^27^. In this approach, which represents an emerging area of research, Gaussian process regression is coupled with functional hypothesis testing to detect mutations in oligonucleotide sequences^28–30^. This method prevents the inclusion of confounding variables in the model and reduces the chances of overfitting. Both these advances (XAI and hypothesis-testing) are powerful ways to address issues with validation of dimensionality reduction and “black box” methods by offering ways to understand the classification and verify its chemical soundness.

This combination of ML and intrinsic SERS has also generated new issues -or questions- regarding SERS data. (1) To achieve good models, ML requires robust and large datasets. Achieving such datasets can be challenging with real-world SERS data. Specifically, the important SERS features can be hidden in the complex and varying background signal, which must be eliminated prior to carrying out the ML analysis. To this end, pre-processing of the data has key importance to ensure that good Raman—molecular—information is uniquely used in the models. SERS data can be processed by removing broad background signal and noise from spectra. However, results can vary between spectra within a dataset making pre-processing of Raman data hard to scale-up to large datasets. In recent years, automated platforms for pre-processing Raman data have been developed^31–33^. (2) How models are validated it’s another key question for SERS + ML. Specifically, auto-validation (validation within the training set) should be avoided even with smaller datasets as it most likely produces overfitted models. Cross-validation for SERS + ML data can be performed with a training and test set (80/20 or 70/30 split) using k-fold (5- or 10-fold) cross-validation, which has the additional benefit of providing confidence intervals for accuracy. (3) As mentioned above, the interpretation of discriminative ML models is often a challenge. Complex models that combine dimensionality reduction with non-linear classification algorithms are generally considered a “black box”, not permitting human comprehension of how data are classified within the original data space. However, for SERS, interpretation also becomes an opportunity thanks to the molecular information directly connected with SERS bands. Specifically, XAI and ML methods that explicitly specify the features responsible for the model classification can be powerful in offering molecular information that can be used to convince final users of the trustworthiness of a model, as well as to generate new chemical insight, such as unexpected differences between sample classes that can then be further explored.

### Current uses of machine learning in **extrinsic** SERS sensing

In extrinsic SERS sensing, ML can be used to extract the sensor reporter signal from a complex background or from a complex mixture of reporters in the case of multiplexing. This approach offers improved sensitivity and can boost multiplexing capabilities of this sensing principle. It’s important to note that this strategy is a significant deviation from intrinsic SERS sensing, because in extrinsic SERS the significant part of the spectra (signal for analyte detection) is well-known because it comes from the chosen Raman reporter. In the case of extrinsic sensing, ML can boost some figures of merit but does not significantly change the analytical method, unlike for intrinsic sensing where ML can be used to identify novel chemical signatures. Nevertheless, ML has been applied to extrinsic SERS sensing with interesting results.

**Fig. 3:**
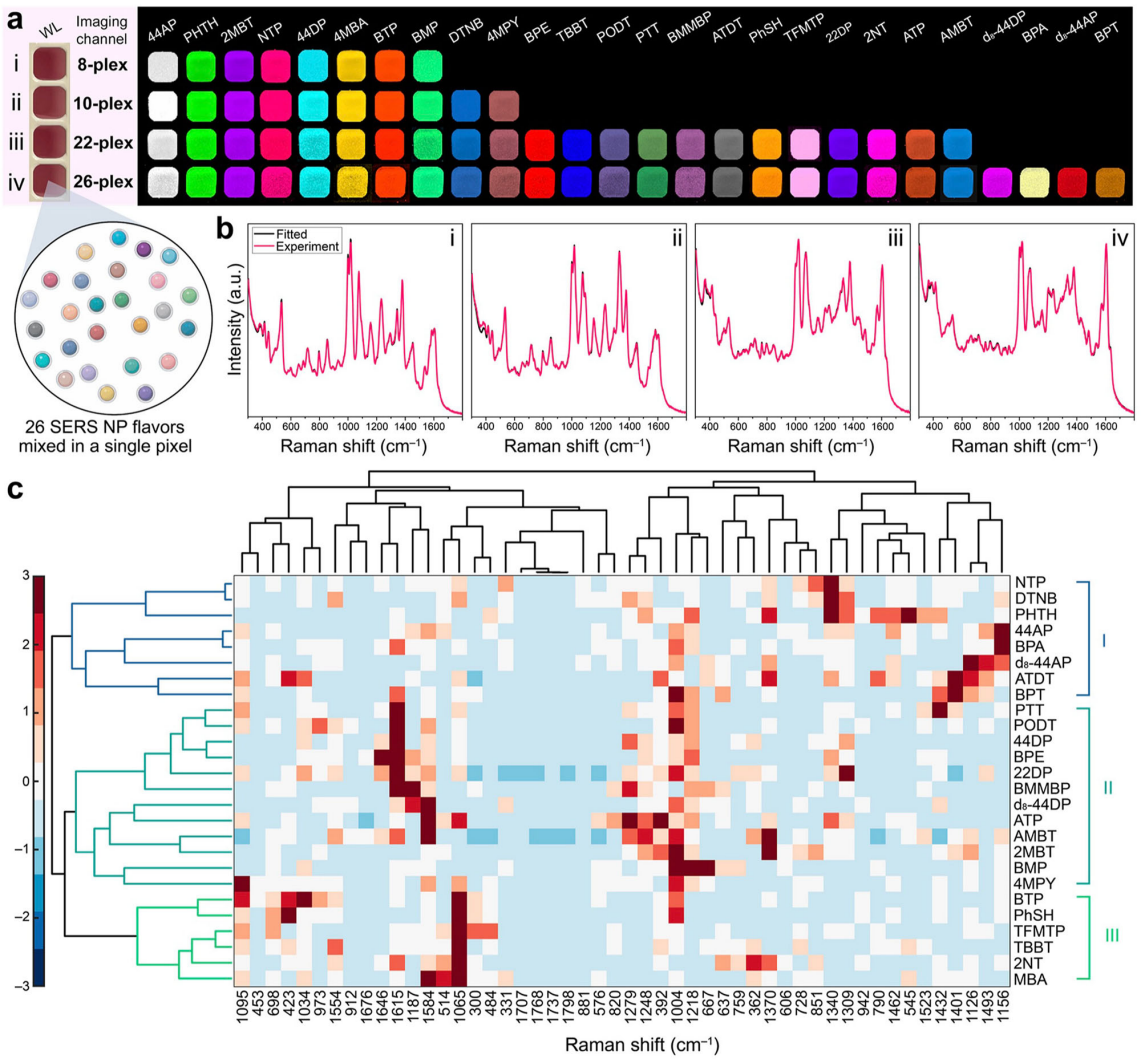
Application of hierarchical clustering to classify the spectra of 26 separate Raman labels (26-plex) of SERS-tags, based on unique spectral features and differences. **a** Multiplex examples with relative reporters. **b** Spectra for multiplex combinations in (**a**). **c** Schematic representation of hierarchical clustering results. Adapted with permission from ref. ^34^ copyright 2022 American Chemical Society.

An example of ML used to boost multiplexing is the work by Eremina et al. where a record 26-plex of SERS reporters was achieved^34^. This work demonstrates the use of ML to expand the number of simultaneous Raman reporters detected from 10 (detectable without ML support) to 26. Specifically, hierarchical clustering was used to determine spectral similarities and then to form “families” of reporters. These families were found to cluster together reporters that share structural similarities. Next, non-negative least squares (NNLS) regression and hierarchical clustering were used to identify the combination of all 26 reporters. This approach was demonstrated in SERS imaging of the SERS-tags in noninvasive liver imaging. Specifically, Fig. 3 shows the multiplexing with example spectra, as well as the use of hierarchal clustering to determine spectral similarities and then form “families” of reporters. ML was also recently used to boost multiplexing in reagentless SERS sensors comparing the performance of three different ML techniques on a four-sensor multiplexed mixture^35^. Importantly, unlike for the SERS tags that can be fabricated with any Raman reporter, the pool of reporters available in DNA-based sensors is limited. The available reporters are dye molecules that can be bound to DNA and often have similar structures, making resolving spectra a challenge. In this report, three techniques, a convolutional neural network (CNN), support vector regression (SVR) and XGBoost were compared in analyzing the sensor data after non-negative matrix factorization (NMF)^35^. The group found that CNN could predict with the highest accuracy the specific reporter at a reduced computational cost. The combination of NMF and ML can improve demultiplexing capabilities, enabling 4-plex assays in reagentless sensors working with functional nucleic acids.

### Inverse design and SERS sensing

More recently, ML approaches have been proposed to perform inverse design to benefit SERS sensing. The idea of inverse design is to start with the desired properties (input) and use a computer model to identify the ideal material (output) with the appropriate properties^36^. The model for inverse design is built via ML by producing or analyzing a dataset of materials with defined properties. This method is in contrast with the current way materials are optimized by exploring a set of candidate materials to identify the material with optimal properties. Inverse design is a virtual exploration of the design/properties space. To produce effective and accurate models, data libraries must be large and reliable, which often requires automation for material synthesis. Additionally, inverse design is particularly useful for large design spaces (i.e., when a large number of property-altering modifications are possible within the system), which includes inorganic materials with modifiable elemental composition, organic molecules with modifiable structures, nanoparticle with modifiable shapes, and for DNA molecules with DNA sequences^37–40^. Among many examples, this method has been used to identify optimal inorganic materials and synthetic molecules/routes^37,41^.

**Fig. 4:**
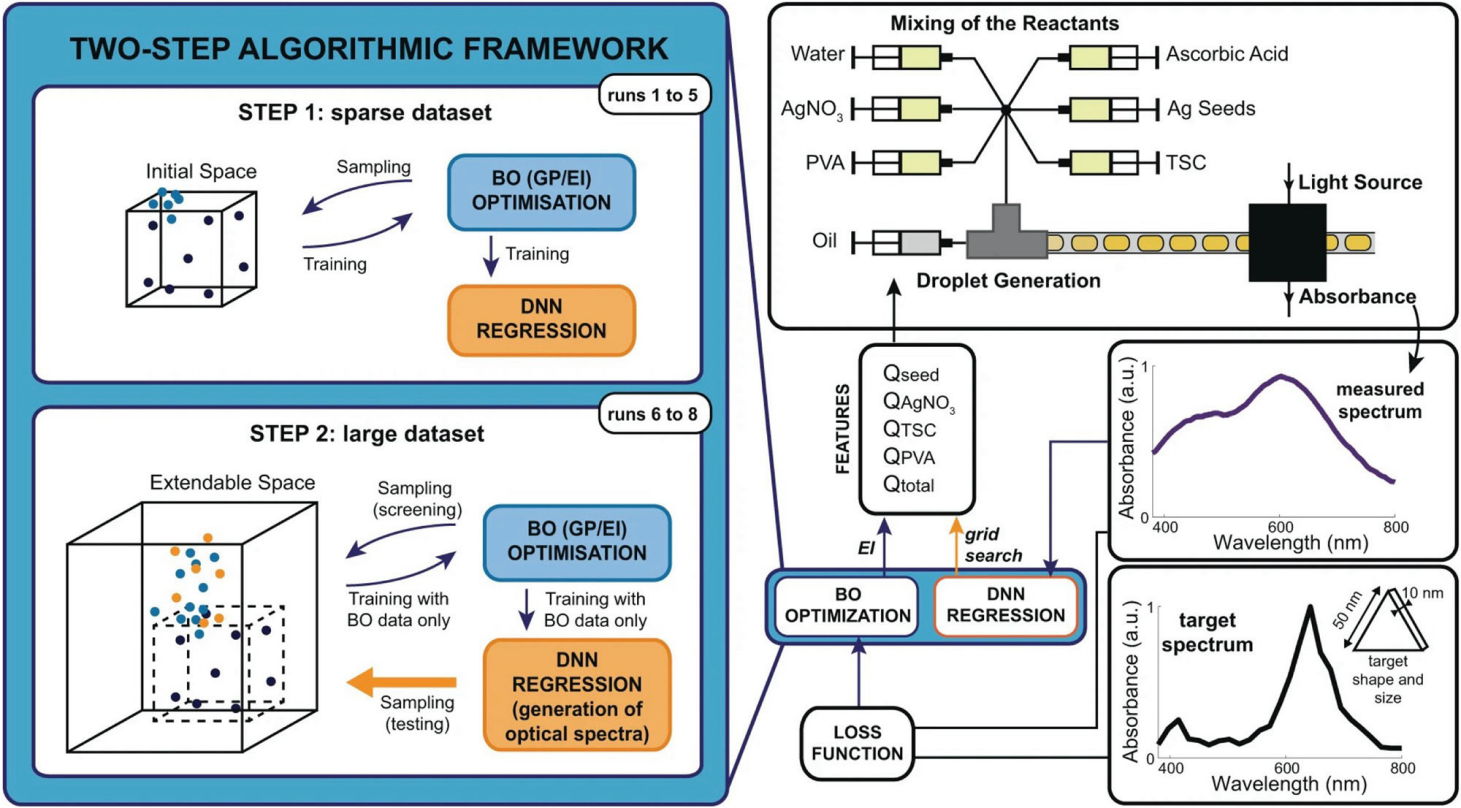
Automated flow synthesis of silver nanoparticles optimized using deep neural networks (DNN) to identify optimal flow parameters to achieve a target spectrum. Adapted with permission from ref. ^42^ copyright 2021 Nature Publishing Group.

In the context of SERS, inverse design for SERS materials has been recently proposed in a review discussing the use of ML for nano-plasmonic materials, discussing the use of ML to tailor the nanostructure morphology to target desired properties (e.g., SERS enhancement)^11^. To this end, nanomaterials have to be produced using automated synthesis. A flow-based synthesis for NP coupled with ML has been recently proposed^42^. In this work, researchers automated the synthesis of silver nanomaterials using flow chemistry and analyzed the design space composed of flow rates of the reagents based on the achieved localized surface plasmon. Figure 4 shows the workflow of using flow-based synthesis of NP coupled with ML. Recently, researchers have also designed a semi-automated system for the synthesis of gold nanoparticle, using robotic injections of the reagents^43^. Advances -as the ones described here- in the automation of synthesis of materials relevant to SERS will enable researchers to build libraries of SERS materials and models based on the SERS performance of materials. These next steps will bring inverse design to the field of SERS sensing, with the potential discovery of new powerful materials. In colloidal synthesis of nanomaterials, as in the advances described above, the fabrication setup is straightforward to automate and was therefore explored first; however, once this approach is demonstrated for SERS substrates, we expect ML-assisted fabrication to be extended to more complex solid-state fabrication techniques (e.g., nanolithography), as already seen in related fields^44^.

### Prospective uses of machine learning in SERS biosensing

As previously mentioned, inverse design is a powerful tool that has been extensively utilized in the design of inorganic and synthetic materials. However, the extension of this concept to biological materials and receptors has not been fully explored. Ideally, the concept of inverse design is well suited to be used to engineer biomaterials and macromolecules, which are based on well-defined repeatable units. For the field of SERS biosensing, this approach could benefit extrinsic sensing by aiding the design of optimal receptor and sensing mechanisms. For this approach to be effective, it requires extensive libraries of receptors with related sensing performance indicators (e.g., k_d_ or limit-of-detection). Such libraries are not readily available for many SERS biosensors due to the low scalability of these sensing platforms. Similarly, as for SERS substrates, this field needs to incorporate automated processes to produce libraries ideal for inverse design. Some recent advances have explored automation in SERS biosensing, machine learning models of biological materials and inverse design of receptors. Notably, generative AI has also been used in this new research direction, with receptor sequences generated based on ML models. This advance has interesting parallels with AI application for text generation with large language models (LLM), which can be applied to biopolymer sequences (e.g., peptides or nucleic acids) in the context of biorecognition. We will give an overview of this exciting research landscape.

**Fig. 5:**
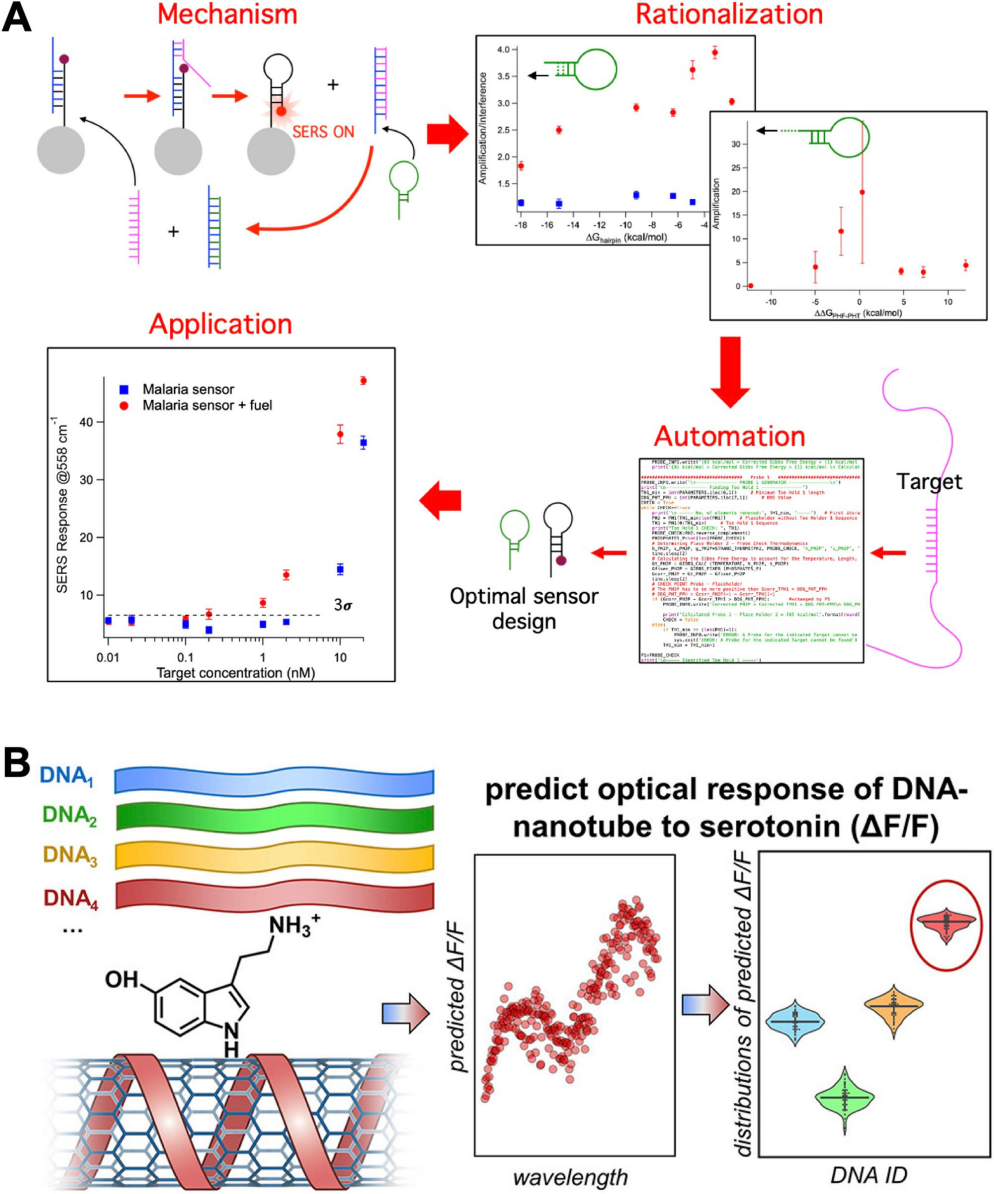
Automated and inverse design of biosensors. **A** A catalytic sensing mechanism for SERS biosensors was first optimized through rational design. The resulting parameters were used to develop an algorithm to automate sensor design and applied to specific sensing applications. Panel (**A**) adapted with permission from ref. ^51^ copyright 2023 American Chemical Society. **B** Sensors based on SWCNT coated with DNA for the detection of serotonin were optimized using ML and inverse design. Libraries of DNA sequences with known responses to serotonin were used to build a model and predict highly responsive sequences. Panel B adapted with permission from ref. ^59^ copyright 2024 American Chemical Society.

The most successful and visible example of using generative AI for biomaterials is the development of AlphaFold^45^. AlphaFold uses an artificial neural network to build a model that maps amino acid sequences to protein structures. To achieve this result, datasets from Protein Data Bank (PDB)^45,46^ including sequences and experimental structures of 350k of proteins were used for training. The inverse approach (predicting the sequence necessary to better fit mutating proteins) has been used in adapting antibodies to viral mutations, showing how these methods can be integrated for the inverse design of receptors^47^. In the biosensor space, the development of generative AI models represents an emerging direction of research that holds the promise to improve over discriminative models. Current examples of generative approaches in this field focus on using the nucleotide sequence as input. Random DNA libraries were used to study the off-target effect in toehold-mediated strand displacement, to simulate interferences expected in vivo. This work identifies the features that are correlated to the most robust sensors using regression trees^48^. In a series of studies, the efficiency of libraries of RNA switches were studied using deep learning^49,50^. Interestingly, in one study thermodynamic features were not pre-assigned but rather the RNA sequence was used directly in the ML model. This approach revealed that sequence is a (10-fold) better predictor of the efficiency of RNA switches than thermodynamic and kinetic features combined^49^. A separate, but complementary, study used a language model (LM) approach to design optimized toehold switches^50^. The LM interprets the toehold sequence as a text sentence formed by k-mer “words” and uses an encoder to map the words into vectors. A decoder, which performs the inverse mapping, then enables the generation of new sentences corresponding to the improved toehold switches. These findings open a new interesting opportunity for the field of AI-driven biosensors, which could benefit from the use of language models rather than limiting model correlation to a few thermodynamic features. Similarly, as for inverse design of materials -discussed above-, these approaches will require large biosensor libraries, which are currently hard to produce. In this context, our lab has recently developed an algorithm to automate the sensor design of a SERS biosensors that uses toehold displacement and DNA catalysis in its sensing mechanism^51^. In this work, we optimize the thermodynamic parameters of a reagentless SERS biosensor to generate an automated design algorithm (as displayed in Fig. 5A). The goal is to remove the trial-and-error phase in the biosensor design workflow to help generate datasets appropriate for AI-assisted design.

Potential benefits of using generative approaches for biosensing applications are also highlighted by the recent work of Sadeghi et al. on the design of DNA-stabilized silver nanoclusters (Ag_N_-DNAs)^52^. These nanoclusters, which comprise 10–30 silver atoms stabilized by one or two single-stranded DNA oligomers, are tunable fluorophores controlled by the DNA oligomer sequence. Discriminative ML approaches have been successful in designing Ag_N_-DNAs by mapping the DNA sequence onto a nanocluster property, such as the emission color, and by using ML chemistry-based classifiers^53–57^. Nevertheless, these approaches are restricted to using a single property and they require additional structural insight to define the ML features. The alternative approach of Sadeghi et al. circumvents these limitations by using a variational autoencoder model that maps the DNA sequence to multiple nanocluster properties and enables the design of Ag_N_-DNAs through a generative model. The VAE approach uses only the DNA sequence as input, thus avoiding the need for structure-based features in the nanocluster design.

A recent and -to our knowledge- the only example of sensing receptor optimization via ML for biosensing was the use of DNA-based receptors in single-walled carbon nanotubes (SWNT) sensors for neurotransmitters^58,59^. In these sensors, SWNT are functionalized with DNA, which affects the optical properties of the nanotubes. Specific DNA sequences are selective for small molecules and can bind them by changing SWNT-DNA interaction. In this work, researchers used a library of ≈ 100 DNA sequences selected from a larger pool of sequences based on their binding properties. The sequences were modeled using a support vector machine (SVM) for response to serotonin and for optical response (measured as signal change). This workflow is displayed in Fig. 5B. The modeled sequences were then validated experimentally discovering five DNA−SWNT sensors with higher fluorescence intensity response to serotonin than obtained previously. This work demonstrates how DNA sequences can be predicted using ML approaches to optimize DNA-based sensors -including SERS biosensors-.

Finally, deep-learning-assisted algorithms are also emerging to solve the complex spectrum-structure correlation in SERS data, as discussed in-depth elsewhere^60^. In brief, ML models and large datasets can be used to generally solve the problem of what specific molecular features are responsible for specific Raman and SERS data. The new tools can help improve library matching (identify a compound across a library of spectra) and, more interestingly, can also be used for de novo molecular generation (i.e., generate a predicted structure based on a spectrum-generative AI in the context of intrinsic SERS-). An interesting example of the latter is Ramancloud, an online platform with the goal of democratization of AI for spectrum analysis^31^. Within the platform it is possible to analyze SERS spectra and solve both spectrum-structure and structure-spectrum problems with AI-based tools.

### Conclusion and prospective

In conclusion, the significant impact that ML has and will continue to have on SERS biosensing has become increasingly clear. In the context of intrinsic SERS, ML is used to decode complex datasets in SERS-based classification of biological samples. SERS data is ideally suited for this task due to sharp features associated with specific molecular information. Recent advances in this sub-field have focused on the interpretation of the classification model in order to increase confidence in the models and to use ML to gain new chemical insights. In extrinsic SERS, ML has been used to expand the multiplexing capabilities, increasing the reporters detectable simultaneously to take full advantage of a key feature of SERS biosensors. These ML-driven improvements allow SERS biosensing to become a more reliable tool overall, offering new and improved diagnostic capabilities for a broad range of targets. The remaining challenges and opportunities in this sub-field concern the ability to produce robust and large SERS datasets, as well as to ensure model validation and generalizability.

In related fields to SERS sensing (material science and biochemistry), inverse design is emerging as an ML-powered tool to change the way we discover new inorganic and biomaterials. We recently saw several examples of using inverse design for bioreceptors and SERS materials, but significant work and opportunities are still available in this space. The shift that we could see in SERS biosensing could be similar to what has happened in ML for language, which has evolved from understanding language (e.g., Siri or Alexa) to generating language (ChatGPT). We could soon witness the power of generative AI emerging for SERS sensors even as discriminative AI continues to improve SERS sensing.

## Data Availability

No datasets were generated or analyzed during the current study.
